# Mapping Molecular Agents Distributions in Whole Mice Hearts Using Born-Normalized Optical Projection Tomography

**DOI:** 10.1371/journal.pone.0034427

**Published:** 2012-04-11

**Authors:** Claudio Vinegoni, Paolo Fumene Feruglio, Daniel Razansky, Rostic Gorbatov, Vasilis Ntziachristos, Andrea Sbarbati, Matthias Nahrendorf, Ralph Weissleder

**Affiliations:** 1 Center for System Biology, Massachusetts General Hospital and Harvard Medical School, Richard B. Simches Research Center, Boston, Massachusetts, United States of America; 2 Center for Molecular Imaging Research, Massachusetts General Hospital and Harvard Medical School, Charlestown, Massachusetts, United States of America; 3 Department Neurological, Neuropsychological, Morphological and Movement Sciences, University of Verona, Verona, Italy; 4 Institute for Biological and Medical Imaging, Technical University of Munich and Helmholtz Center Munich, Neuherberg, Germany; Maastricht University, Netherlands

## Abstract

To date there is a lack of tools to map the spatio-temporal dynamics of diverse cells in experimental heart models. Conventional histology is labor intensive with limited coverage, whereas many imaging techniques do not have sufficiently high enough spatial resolution to map cell distributions. We have designed and built a high resolution, dual channel Born-normalized near-infrared fluorescence optical projection tomography system to quantitatively and spatially resolve molecular agents distribution within whole murine heart. We validated the use of the system in a mouse model of monocytes/macrophages recruitment during myocardial infarction. While acquired, data were processed and reconstructed in real time. Tomographic analysis and visualization of the key inflammatory components were obtained via a mathematical formalism based on left ventricular modeling. We observed extensive monocyte recruitment within and around the infarcted areas and discovered that monocytes were also extensively recruited into non-ischemic myocardium, beyond that of injured tissue, such as the septum.

## Introduction

The understanding of many dynamic processes has been hampered by a lack of appropriate three-dimensional high-resolution imaging tools. Over the last two decades, microscopy has rapidly advanced, now achieving high spatial and temporal resolutions [1], greater penetration depth [2], multi-reporter visualization, *in vivo* imaging capability [3], and the capacity for digital distribution analysis [4]. However, a prevailing problem with conventional sectioning, visualization and subsequent image fusion [5], has been the inordinate amount of time required to obtain three-dimensional information at the whole organ level. While this approach is feasible for small organs, large blood-rich, optically non-transparent organs such as the heart have been more difficult to characterize.

To date, optical tomographic approaches have been successfully used to image whole animals, albeit at a low spatial resolution [6]. Meso [7]- and microscopic tomographic techniques such as light sheet microscopy and optical projection tomography (OPT) have also been described [8], [9] and they typically rely on the use of optically transparent model organisms such as zebra fish and embryos, or ex-vivo chemically cleared samples, and use fluorescent proteins or antibody staining as optical contrast markers [10]. Imaging of non-transparent and blood-rich tissues such as the heart, has been far more challenging. We reported on a complementary approach termed Born-normalized [11] fluorescence projection tomography where contrast is obtained through differences in optical properties. Herein we extend this modality to multiple fluorescence channel acquisition in combination with the use of different image processing and reconstruction algorithms and through the implementation of mapping projection tools for high-content tomographic data analysis of molecular probes for cardiac research. This makes the described technique uniquely suited to quantitatively and spatially resolve the recruitment within the whole heart of phagocytes based on the uptake of CLIO-VT750 nanoparticles, and cathepsin B protease activity based on cleavage of Prosense-680, in order to better comprehend their spatio-temporal distribution. In view of the growing number of biocompatible molecular probes for in vivo use we anticipate that this technique will have considerable applications not only for biomedical research but possibly also in clinical pathology laboratories.

## Materials and Methods

### Experimental Setup

**Figure 1:**
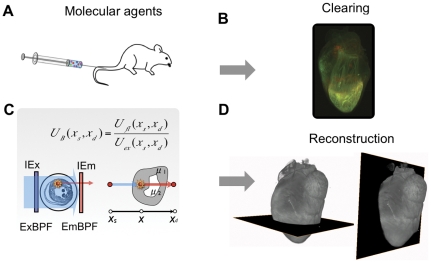
Born-normalized molecular optical projection tomography of inflamed hearts. (A) Dual channel imaging. A cocktail containing two different molecular imaging agents is administered intravenously. (B) Dual channel Born-normalized near-infrared transillumination fluorescence image on day 5 after myocardial infarction (MI), 24 hours after probe injection. High signal in both the 680 nm (left) and 750 nm (right) fluorescence channels can be observed in the heart region, indicating elevated protease and phagocytic activity. (C) A narrow bandwidth light source excites the fluorophores attached to a molecular probe located within the sample, and fluorescent light is then emitted. Both excitation and emission light are, in part, absorbed by the sample on the way to and from the fluorophore, respectively. Using a Born-normalized approach, fluorescence tomographic reconstructions can be obtained, after correcting for the sample's absorption map. (D) Image processing algorithms are applied to tomographically obtain signal distribution reconstructions in whole hearts and to remove noise and artifacts contributions.

**Figure 2:**
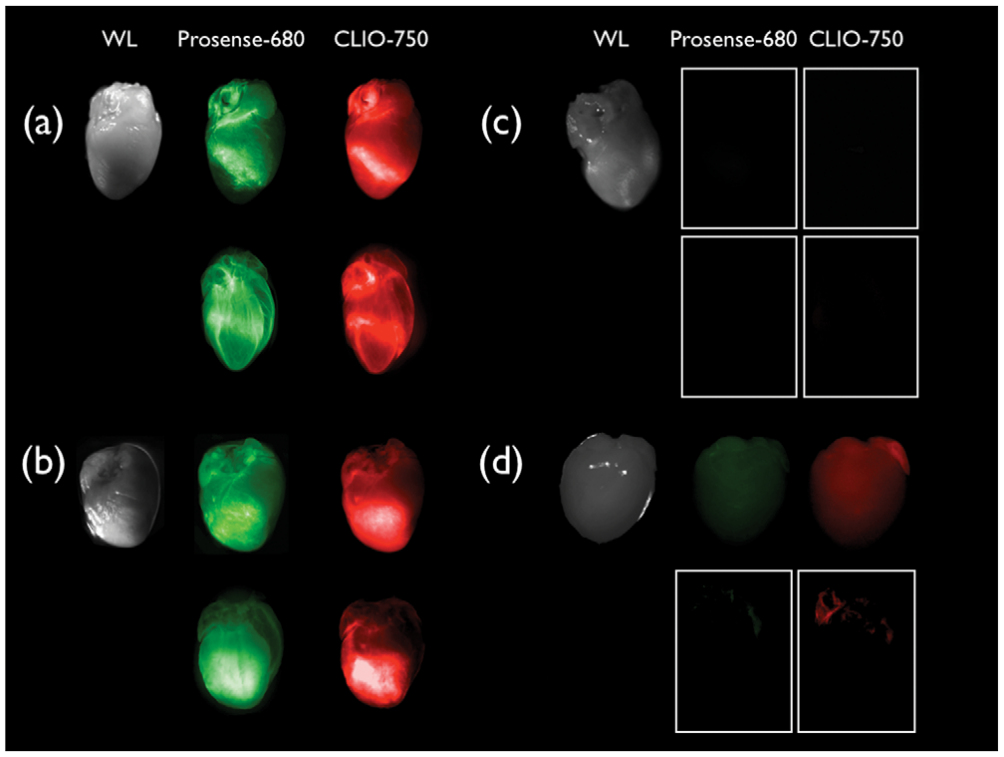
Fluorescence Reflectance Imaging. White light and ex-vivo fluorescence reflectance imaging (FRI) in the VT-680 and VT-750 fluorescence channels evidence Prosense-680 and Clio-750 activities on the hearts' surface at day 5 post-MI. Probes were injected 24 h prior to heart explantation. (A) Myocardial infarct (permanent occlusion of coronary artery), probes injected. FRI images reveal strong localized near infrared fluorescence (NIRF) signal. (B) Ischemia reperfusion injury, probes injected. FRI images indicate strong (NIRF) signal homogeneously distributed in the whole anterior left ventricle. (C) Myocardial infarct, no probes injected. Extremely weak autofluorescence signal is present in the FRI images. (D) Heart without myocardial infarction (sham surgery), probes injected. Weak NIRF signal homogenously distributed in the whole heart is present. For each fluorescence channel all FRI images are acquired using the same parameters and identically scaled. In each panel the lower row shows the corresponding Born normalized projection for the cleared sample.

**Figure 3:**
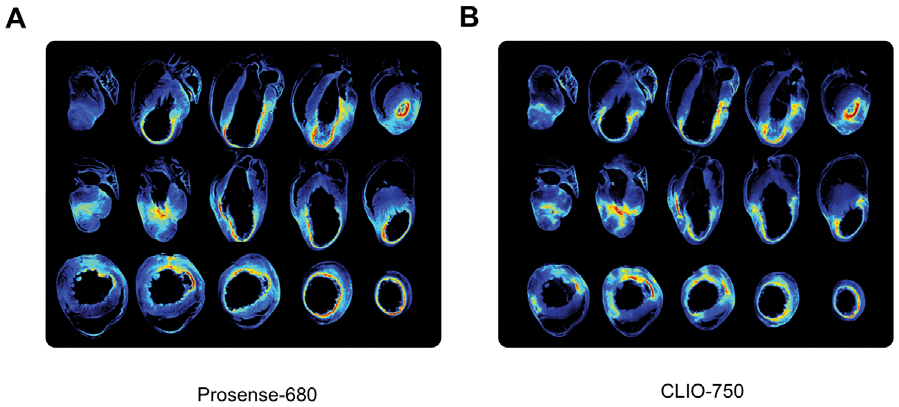
Tomographic reconstructions. Coronal (top), saggital (middle), and axial (bottom) tomographic reconstructions of enzyme (A) and phagocytic cell (B) distributions. After permanent occlusion of the coronary artery, both probes are primarily confined to the infarct scar area. This area is associated with a strong localized near-infrared fluorescence (NIRF) signal, whereas only weak fluorescence intensity is observed in the basal part of the ventricle.

**Figure 4:**
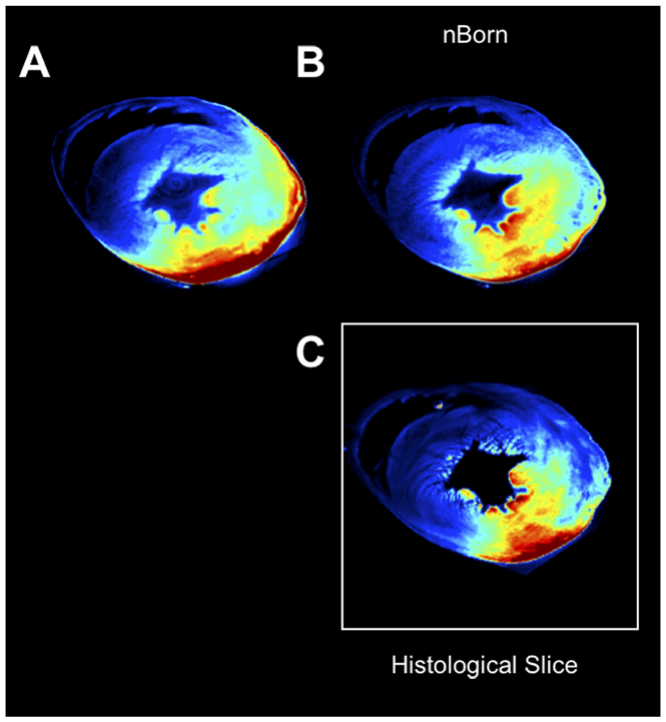
Validation of tomographic reconstructions. Comparison between OPT reconstructions and histological sections of molecular probe activity distribution (Prosense-680) in the inflamed heart after MI. Comparisons between reconstructions obtained with fluorescence OPT (A), Bornnormalized OPT (B), and the corresponding histological section (C). Born normalization preserves the molecular distributions in the reconstructed fluorescence channels. This is particularly evident for the papillary muscles located deep within the left ventricle, which appear less fluorescent without normalization, and the epicardium, which always shows bright fluorescent signal in absence of normalization. Born-normalized OPT reconstructions were obtained on the whole heart. The histological section (500 µm) belongs to the same specimen.

**Figure 5:**
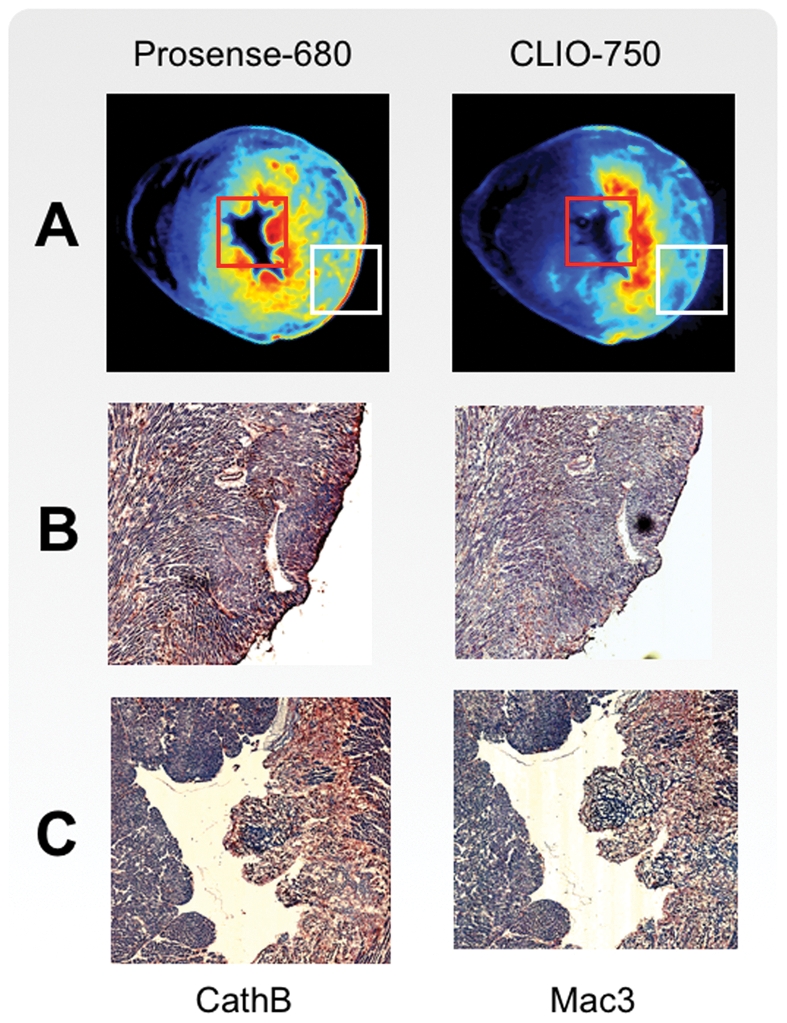
Histological comparison. Born-normalized fluorescence OPT reconstructions (A) of the two molecular imaging agents Prosense-680 and CLIO-750 are compared with immune-reactive staining of the imaging target protease Cathepsin B (B) and for the Mac3 macrophage antigen (C).

The imaging system consists of a modular, home-built setup, capable of acquiring multichannel transillumination images from both fluorescence and attenuation data (Text S1, Figure S1). Spectrally filtered white light (e.g. at 680 nm, 750 nm as well as multiple channels in the visible-NIR optical spectrum for multispectral absorption mapping) was used to acquire intrinsic transillumination images as well as normalization absorption maps at relevant excitation wavelengths. For low scattering conditions such as in cleared hearts, a filtered backprojection (FBP) algorithm was used to compute the absorption map 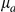
(*x,y*). Because absorption has a spatial non-uniform pattern, the distribution of molecular probes could not be directly obtained with the inverse Radon approach through a FBP method. Instead, it was necessary to solve the inverse problem using accurate knowledge of the optical absorption distribution within the heart (Figure 1C). We therefore used a Born-ratio weighting method with a normalized transillumination approach. Since the molecular probes were excited in the far red and near-infrared range, tissue autofluorescence was negligible (Figure 2C–D) which resulted in excellent channel separation, and bleaching was tremendously reduced. Due to artifacts introduced by time-dependent intensity fluctuations in the illuminating sources, all projections and Born-normalized data were corrected prior to FBP (Figure S2). Both non-linearity and drift response in CCD detectors can contribute to ring artifacts thus degrading the quality of segmentation algorithms. Sinogram filtering, in combination with a noise reduction algorithm, based on block matching three-dimensional filtering, was therefore implemented (Figure S3). To minimize the reconstruction time, we have implemented the algorithms on a NVIDIA Tesla C2050 graphic card. This allowed us to improve reconstruction times by ∼3000-fold respect to CPU implementation. Figure 3 shows representative examples of consecutive sections taken in axial, coronal and sagittal planes. These planar reconstructions (slice thickness: 10 µm) are useful for inspecting multichannel molecular information at high spatial resolution (70 microns). Validation of the tomographic reconstructions of the probes distribution was obtained by direct comparison with fluorescence reflectance images obtained on adjacent sections (Figure 4). Comparison between Born-normalized fluorescence OPT reconstructions of the two molecular imaging agents with the immune-reactive staining of the imaging target protease Cathepsin B and for the Mac3 macrophage antigen is presented in Figure 5 and shows good agreement between the two.

### Experimental Protocol

A scheme illustrating the experimental protocol is given in Figure S4. A total of 12 mice (Jackson Labs, Bar Harbor, Maine) were used in this study. Myocardial infarction (MI) was induced by left coronary artery ligation. Mice were intubated and ventilated with isoflurane supplemented with oxygen (isoflurane 1% to 2% v/v +2L O2) and a thoracotomy was performed in the fourth left intercostal space. The left ventricle was visualized by inserting a retractor between the ribs and a permanent ligation was performed using an 8.0 nylon monofilament suture on the left anterior descending (LAD) artery at the position of emergence from the left atrium.

The procedure gave rise to a large myocardial infarction (off-white coloring) involving the anterolateral, posterior, and apical regions of the left ventricle. In a subset of mice (N = 3) the ligation was removed after 30 minutes to induce ischemia reperfusion injury. Sham surgery (N = 2) involving thoracotomy without LAD ligation was performed as a control procedure. The Subcommittee on Research Animal Care (SRAC), serving as the Institutional Animal Care and Use Committee for the MGH, approved all studies. The SRAC is accredited with AAALAC and the NIH Office of Laboratory Animal Welfare (A3596-01).

### Molecular probes

Herein we focused on mapping the distribution within the whole heart of two probes: Prosense-680 and CLIO-750 since both probes have been widely studied and validated. Prosense-680 is a commercially available (Perkin-Elmer) protease-activatable fluorescence sensor based on a polymeric scaffold that allows imaging of Cathepsin B activity. CLIO-VT750 is a magneto-fluorescent dextran coated nanoparticle with fluorochromes emitting light at 780 nm and is efficiently ingested by inflammatory phagocytes. Both imaging agents are well validated, their co-localization with the target cell/target molecule has been vetted and flow cytometry and histology studies have shown colocalization of cross linked iron oxide nanoparticles with monocytes and macrophages [12]–[16]. The used protease sensor has also been previously validated [6], [17], [18].

### Sample Preparation

4 days post MI, mice were injected via tail vein with 5 nmol of two imaging agents (Prosense-680, Clio-750) injected separately. After 24 hours (day 5) mice were imaged in-vivo with a fluorescence molecular tomography (FMT) system used in transillumination mode to assess inflammation status. Mice where then anesthetized with an intraperitoneally (IP) injection of a mix of ketamine (90 mg/kg) and xylazine (10 mg/kg) and ventilated. 50 U of heparin were injected IP and a longitudinal laparotomy was performed. The thorax was opened and fluorescently labeled (FITC) microbeads were injected in vivo in the left ventricle. After 5 minutes the inferior vena cava (IVC) was cut distal from the position where 20 mL of saline were infused. After flushing of the whole blood volume, the thorax was opened and the heart removed. Hearts were fixed for 3 hours in PFA, washed in PBS for 15 minutes, embedded in a 0.8% agarose cylinder, and then dehydrated in ethanol for 24 hours. The samples were then transferred in a solution 1∶2 of Benzyl Alcohol and Benzyl Benzoate for the time necessary for the sample to clear (approx. 48 hours).

### Heart Modeling and Segmentation

Once the molecular probes' distributions have been tomographically obtained via Born-normalized fluorescence optical projection tomography, there was a necessity to provide the correct tools to properly describe and characterize their patterns activities within the heart. Ligation of the LAD resulted in large myocardial infarction involving the anterolateral, posterior and apical regions of the heart leading the inflammation to extend and reach to the septum. This allowed to substantially simplify the problem by limiting our study to the heart's left ventricle which is also frequently the site of infarction in patients. Several well-established methods are usually adopted in order to facilitate its segmentation in particular for finite element studies, such as the area length method [19]. The common strategy behind all these approaches is based on the fact that the LV geometrical shape can be reduced to a much simple shape and modeled as a thick-walled truncated ellipsoid [20]–[22]. The simplification introduced with this assumption does in fact greatly reduce the mathematical complexity of the problem without compromising the accuracy since the intrinsic heart geometry reflects such a degree of symmetry. While other shapes such as the sphere or the cone can be used to describe the LV geometry, in particular during the phases of maximum expansion or contraction, for OPT ex-vivo studies a truncated ellipsoid was the choice of excellence. Following the formalism given in [23], [24] we defined a set of prolate spheroidal coordinates 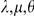 (Figure S5) with coordinate transformation given by:
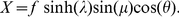
(1)

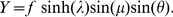
(2)

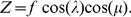
(3)where f is the focal distance of the ellipsoid.

With the use of a semi-automatic segmentation software ITK SNAP [25] the left ventricles of all hearts were segmented, meshed with the use of iso2mesh software [26], and modeled as an ellipsoid following the method as indicated in [24]. The three orthogonal axes of the ellipsoid were first estimated making use of the anterio-inferior and lateral views of the ventricular images [27] and then by fitting the actual external heart surface (epicardium) with the ellipsoidal shell. The Z axes of the left ventricles were fitted with a line using the centroids of the short axis as in [28].

### Molecular Map Projections

Once we found the solution to the best fitting problem of the left ventricle, we took advantage of the geometry of the ellipsoid and the well known theory of map projection in order to visualize the molecular probe distributions in the inflamed LV. While different families of map projections are available, with all of them inevitably introducing a certain degree of distortion, we chose for our model those ones mapping over flat surfaces. In particular we relied on the cylindrical representation. Because the LV is a 3D structure with a non-negligible thickness, different concentric ellipsoids lying within each other could be used to encapsulate and fit the left-ventricular myocardium giving rise to a set of planar maps in cylindrical coordinates, one for each ellipsoid. Instead of plotting a three-dimensional view of this planar set of maps (Figure S5) we opted for a 2D visualization (“2D total projection”), integrating the total signal along the normal radial direction of the ellipsoid (i.e. sum over all the maps). When considering the morphological information obtained from the tomographical reconstruction via absorption OPT, the 2D total projections will then contain information regarding the thickness of the left-ventricular myocardium. For what concerns instead the molecular probes' fluorescence signal representations, these were normalized by the LV thickness. Because the LV was well approximated by an ellipsoid for about three quarters of its maximum axis starting from the heart's apex, the top quarter of the map (the one above the horizontal white line indicated in all cylindrical maps) was not representative of such a modeling even if the signal is shown within this region of the map. All calculations on volumetric probes concentration or colocalization are limited to this area. Bull's Eye representations are considered too.

### Colocalization of Probes

In order to characterize the volumetric distribution of the two molecular probes (Prosense-680, Clio-750) and to determine the strength of their colocalization we implemented the Cross Correlation Function (CCF) [29] (Figure S6). Individually normalized 3D probe distributions were spatially shifted with respect to each other (Δx, Δy, Δz) and the correspondent pixel-per-pixel overlap coefficients (r) were calculated. The obtained coefficients were then plotted in 3D as a function of Δx, Δy, Δz. A perfect probe colocalization leads to a CCF equal to 1 with maximal peak when the two origins are overlapped (i.e Δx = Δy = Δz = 0). A value of CCF equal to 0 means instead that no colocalization between the two probes is present. The full-width at half-maximum (FWHM) of the CCF provides a measure of the spreadness of the signal. Due to the high level of symmetry of the left ventricle, CCF was also explored considering the rotation along the three main orthogonal axes of the fitting ellipsoid. Since considering all six possible degrees of freedom simultaneously is computational demanding we used a suboptimal solution limiting the exploration of CCF for rotational shifts restricted for the case of the center of rotation as the point in which the maximum of CCF is achieved, using translational shifts only. Here CCF was used, instead of a more simple approach based on a single correlation coefficient, because it provides more information regarding the colocalization of the two signals. High CCF's sharpness combined with a maximum at zero-relative translations are all indicators of good spatial correlation.

### Histopathological Validation

Serial adjacent 6 µm thick sections were used for immunohistochemical staining of macrophages (MAC-3, BD Pharmingen, San Diego, Calif), and cathepsin B (sc-6493, Santa Cruz biotechnology, Santa Cruz, Calif) using appropriate secondary antibodies (Figure 5).

## Results

Overall, the experimental approach involves the following steps: a) in vivo administration of molecular probes (or fluorescently labeled cells) and subsequent sample preparation; b) image acquisition; c) reconstruction, post-processing and correlation analysis; and d) data mapping, analysis and visualization (Figure 1, Figure S4). To achieve target specificity, here we administered a cocktail containing different molecular imaging agents intravenously in the live mouse. This not only allows the probes to be homogeneously distributed throughout the entire body, but also enables the use of functional probes (e.g. sensors for enzyme activity) or labeled drugs, which are often difficult to measure in tissue sections prior to animal sacrifice. The injection of such agents also allows for complementary kinetic imaging in living mice [30]. At specific time points after probe administration, animals are then sacrificed, perfused and their hearts removed. Harvested hearts were first dehydrated through a series of alcohol concentrations, and chemically cleared using a 1∶2 benzyl alcohol/benzyl benzoate (BABB) solution, before being imaged in toto.

**Figure 6:**
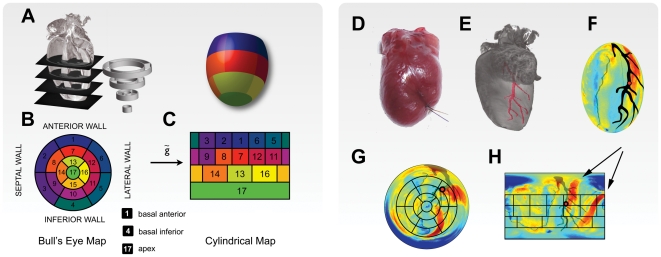
Heart modeling and segmentation. A. Ellipsoid heart modeling for left ventricle segmentation and American Heart Association (AHA)-conform segmentation. Bull's Eye (B) and cylindrical maps (C) are generated from the radial projection of the probes' tomographic reconstructions, over the surfaces of the concentric fitting ellipsoids. The correlation between the 17-sector Bull's Eye map and analogs on the unfolded cylindrical map is given. (D) Myocardial infarction with its corresponding occlusion and visible ligation thread, in the left anterior descending coronary artery (LAD). (E) Tomographic reconstruction of a non-injured heart. The reconstructed LAD (shown in red), courses to the apex of the heart and perfuses the basal anterior, the basal lateral and anterolateral, as well as the apical lateral walls. (F) A segmented left ventricle ellipsoid representation where different colors represent the wall thickness. Map projection (over a flat surface) of the ventricle wall thickness, according to both a Bull's Eye (G) and a cylindrical (H) map representation. Papillary muscles (black arrows) can be clearly identified by their thicknesses. The LAD is mapped and represented in black, while the black grid indicates the location of the 17 sectors. The red spot indicates the location of the ligation.

On average, we obtained ∼500 reconstructed slices per murine heart, for each molecular probe administered in addition to the ones obtained for multispectral absorption imaging (Figure 3). To better navigate these datasets and to visualize differences in probe distribution, we implemented a number of different mapping algorithms as summarized in Figure 6. These are all based on the segmentation of the left ventricle, which can be modeled as a thick walled truncated ellipsoid, and on the theory of map projection. While several families of map projections are available we prefer those that map over flat surfaces. In particular we relied on the cylindrical and the Bull's Eye representations (Figure 6) based on the standardized 17-segment model of the American Heart Association (AHA) [31], which is typically used for nuclear imaging applications. To optimize multichannel whole heart mapping, the above generic methods of visualization tools underwent several iterations of improvement.

The analytical method was subsequently applied for investigations of myocardial infarction, and specifically, to the mapping of inflammatory cell distribution in the whole heart. The currently established paradigm states that circulating monocytes are recruited to the immediate vicinity of the infarcted myocardium [32], where they elicit stage-specific healing during a series of reparative phases [33]. While there have been considerable advances in our understanding of this repair process [33], several important questions remain. For instance, the overall magnitude and spatial distribution of the recruited cells still remains unknown. Perhaps even more importantly, the mechanism by which different monocyte subsets play different roles in tissue destruction and subsequent healing is likewise undetermined. Thus, in a bid to address such questions, we used a mouse model of left coronary artery ligation, where mice were intravenously co-injected with a monocyte-targeting nanoparticle (CLIO-750), a cathepsin B imaging probe (Prosense-680).. The probe cocktail was administered a day before the sacrifice and allowed interrogation of different aspects of innate immune cell biology. Fluorescent microbeads were injected before harvesting to delineate the infarct area.

**Figure 7:**
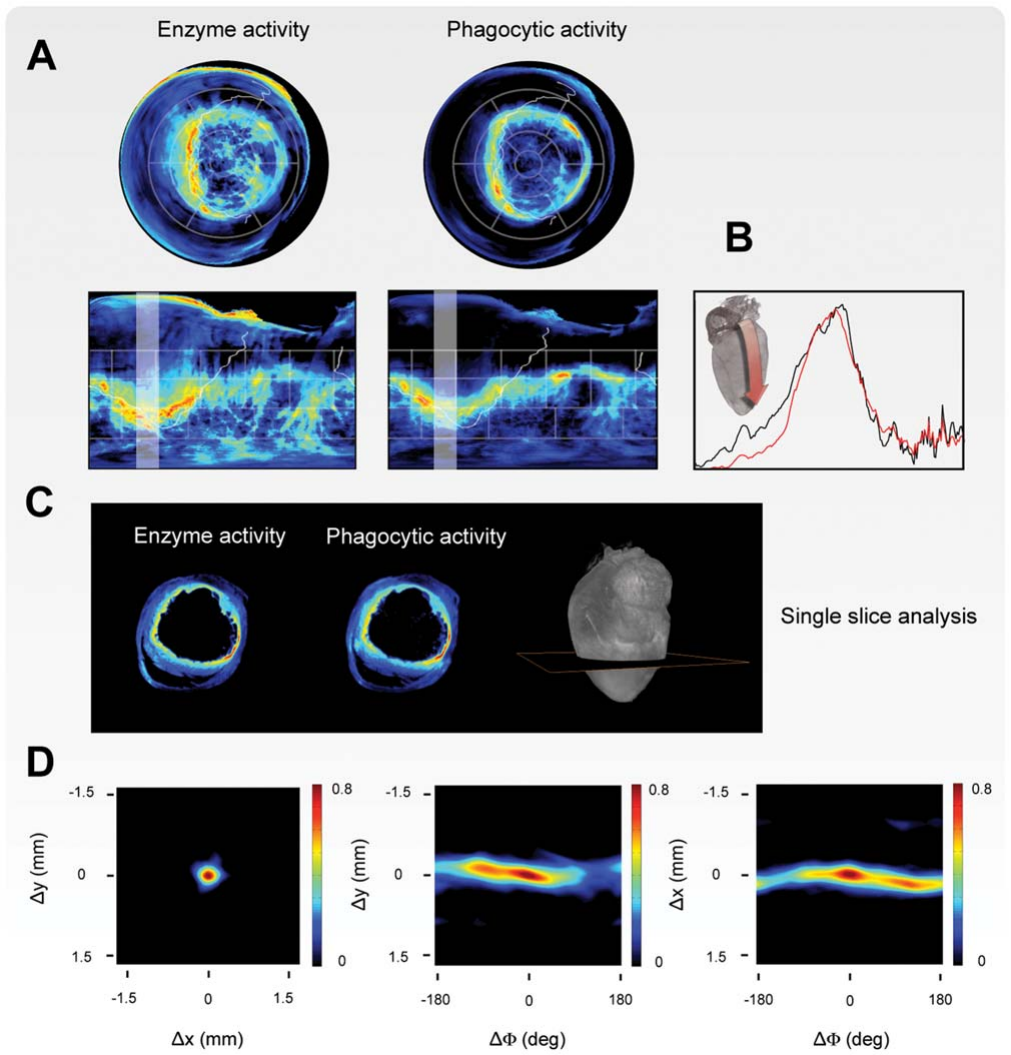
Probe activity distribution maps in inflamed left ventricle. (A) Bull's Eye (top) and cylindrical (right) maps of molecular probe activity at day 5 post myocardial infarction. Inflammation is accentuated along the border of the injured tissue, while the core of the infarct displays low signal. Continuous white line indicates the border of the interventricular septum. (B) One-dimensional plots showing the intensities of the probes activity, along the directions indicated in the maps (white faded bands). Signal plots indicate a high correlation in probe activities. (C) Axial reconstructions of cell-associated probe activity along the indicated plane, with their corresponding co-localization maps. (D) Overlapping cross correlation functions (CCF) are calculated for the two channels, taking into account both translational and rotational shifts. Overall, a high degree of correlation is present, however there are distinct differences in each channel reflecting the different spatial distribution of the molecular target.

As expected, we observed extensive monocyte recruitment within and around the infarcted area. Within 1–3 mm of the infarct periphery the probe distribution per mm^3^ associated with monocytes recruitment was found to be approximately 3 times higher then within 4–6 mm of the infarct with focal peak around 8 times higher. Unexpectedly, however, we discovered that both probes were present in remote areas suggesting that monocytes and phagocytic cells were also extensively recruited to normal myocardium, beyond that of injured tissue (Figure 7A, 7B, and 8A). Indeed, mapping experiments showed that molecular probes distributions extended even into very remote and entirely non-ischemic regions such as the septum. In some animals, the fraction of monocytes recruited to distant normal myocardium and estimated by probe distribution reached as high as 50% of all of monocytes. The presence of recruited cells in these remote locations was subsequently confirmed by much more time consuming immunohistochemistry. These results have led to additional profiling experiments deciphering the specific role of these cells, i.e. recruitment route versus specific local function.

**Figure 8:**
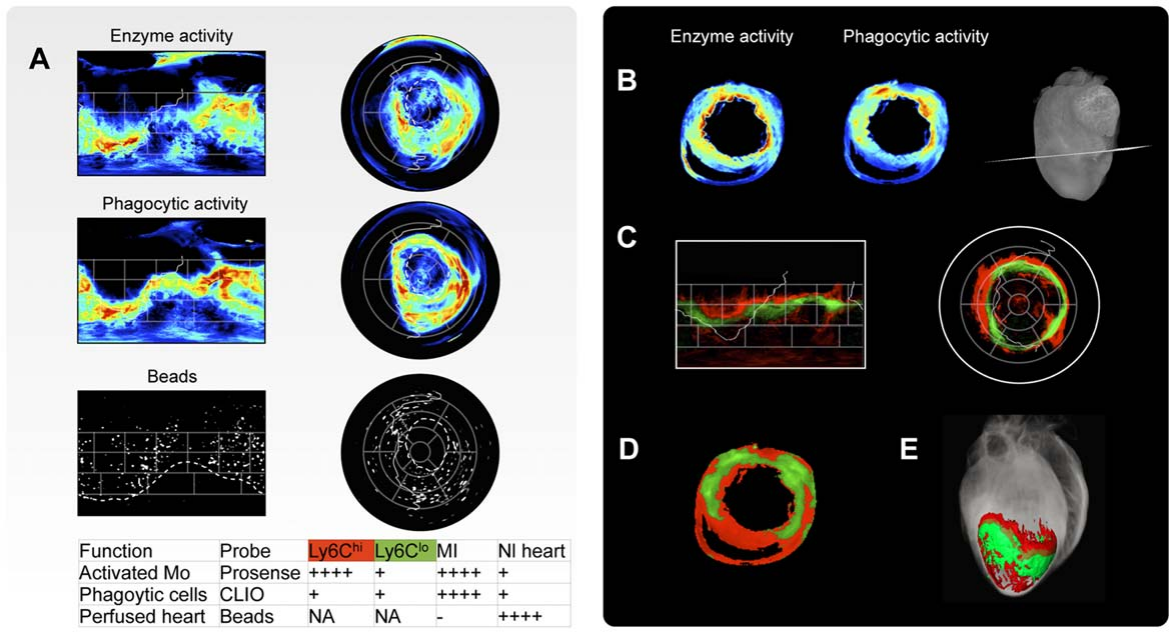
Quantification of probe activity in infarct area. (A) Cylindrical (left) and Bull's Eye (right) maps of molecular probe and bead distribution of an excised heart at day 5 after myocardial infarction. High cathepsin B and phagocytic activity, are evident in the proximity of the injured area with a high degree of colocalization. Fluorescently labeled (FITC) microbeads, injected in vivo into the left ventricle before harvesting, are homogeneously distributed across the whole heart, with the exception of the non-perfused infarct area. The white dotted line indicates the border of the necrotic infarct scar, localized in apical and lateral segments. Continuous white line indicates the border of the interventricular septum. (B) Axial reconstructions of probe activities at the indicated plane. (C) Cylindrical (left) and Bull's Eye (right) representation of reparative (Ly6C^lo^, green) and inflammatory (Ly6C^hi^, red) monocyte subsets together with the an axial (D) and tomographic (E) reconstruction.

Beyond the spatial mapping of cells, functional probes also allow monocyte subtypes to be distinguished. In [12] we have already determined that Ly-6C^hi^ and -6C^lo^ monocytes sequentially enter the infarct and established that these two subsets already commit for specific functions. In that study mice subjected to coronary ligation received intravenous injections of various molecular imaging agents 1–7 d after MI to determine phagocytosis and proteinase activity in vivo. Probes included fluorescent nanoparticles (CLIO-VT680) that are efficiently ingested by phagocytes, and activatable fluorescent sensors reporting either on cathepsin B, L, S (Prosense-680. 1 d later, we have analyzed monocytes freshly isolated from infarcts. We found that both Ly-6C^hi^ and -6C^lo^ monocytes exhibited equal phagocytic capacity in vivo. However Ly-6C^hi^, but not Ly-6C^lo^, monocytes showed high proteinase activity, a process involved in the breakdown of extracellular matrix.

Capitalizing on the work of [12] and the obtained probes distribution maps of both Prosense-680 and Clio-VT750 within the heart, we calculated the ratio between the two in order to find the difference in distribution of the reparative monocyte subsets (Ly6C^lo^) with respect to the inflammatory subtypes (Ly6C^hi^), a ratio above 1 indicating a high concentration of Prosense-680 with respect to Clio-VT750. In the map red color indicates a value of the ratio above 1.1. Green color indicates a value for the ratio between 1.1 and 0.9. Figure 8C shows that reparative (Ly6C^lo^) subtypes were primarily found adjacent to the infarct zone, whereas the Ly6C^hi^ subtype appeared to extend further into the periphery, where it created a “rim” of reactive cells, palisading around the Ly6C^lo^ population. To the best of our knowledge, this has been the first demonstration that monocyte subsets are recruited in a spatially selective manner during myocardial infarction.

## Discussion

In conclusion this study, describes a generic platform for acquisition, mapping, quantification, analysis, and visualization of molecular information in three-dimensions, in both superficial tissues and whole organs. The method is based on advances in Born-normalized near-infrared optical projection fluorescence tomography, and includes filtration algorithms to reduce noise-artifacts, development and analysis of various mapping tools, as well as the fast computation implemented within graphic cards. The method is fast (20 minutes to image a whole murine heart, 5 minutes for the reconstruction), inexpensive (approximately $1,000 for the graphics card and $50,000 for the optical set-up, including the acquisition camera), and versatile. While the focus of this study was post MI inflammation, we envision that this method could also serve to study the organ distribution of stem cells or novel fluorescently labeled drugs, as well as molecular imaging agents. Several enhancements to the current prototype implementing hyperspectral imaging are anticipated, including iterative reconstruction methods, compressive sensing, deconvolution; these should yield even higher resolution maps within shorter time frames. Irrespective of the specific configuration, we believe that the use of near-infrared Born-normalized fluorescence projection tomography for mapping the spatial distribution of cellular and molecular events, will become a useful and indispensable tool for both diagnostic and systems biology applications.

## Supporting Information

Figure S1 **Experimental setup.** (A) WL white light source, SH shutter, BE beam expander, D diffuser, FS optical fiber switch combined with a 680 nm and 750 nm wavelength excitation laser sources, FW ExBPF fast automated filter wheel with band pass filters for both excitation and transmission mode, FW ExBPF fast automated filter wheel with emission band pass filters and neutral density filters, ABS automated beam switcher, S sample, TBPF automated tunable bandpass filter, TL telecentric lens imaging system, CCD imaging camera.
(TIF)

Figure S2 **Power equalization and Born normalization.** Absorption sinogram without (A) and with (B) intrinsic intensity fluctuations corrections. Lamp fluctuations are visible as vertical stripes in (A). (C) Reconstruction after power equalization. Fluorescence sinogram without (D) and with (E) excitation-source intensity fluctuations corrections with relative slice reconstruction (F). (H) Born-normalized fluorescence sinogram after intrinsic and excitation intensity correction with relative slice reconstruction (G).
(TIF)

Figure S3 **Ring artifacts and random noise reduction.** (A) Axial reconstruction, raw data. (B) Reconstruction after post-processing for ring artifacts removal. (C) Reconstruction after ring and noise reduction (by 2D median filter). (D) Reconstruction after ring and noise reduction (by BM3D filter). Artifacts and random noise are corrected on projections, i.e. before backprojection transformation.
(TIF)

Figure S4 **Experimental protocol.** Four days after artery ligation, mice were intravenously co-injected with a cocktail of monocyte-targeting nanoparticle (CLIO-750), and a cathepsin B imaging probe (Prosense-680). After one day fluorescent microbeads were injected in order to delineate the infarct area. Heart was then removed after 5 minutes, chemically treated for 48 hours and imaged. During acquisition data were processed and reconstructed, and finally analyzed.
(TIF)

Figure S5 **Bull's Eye representation.** Cylindrical (A) and Bull's Eye (B) coordinate transformation. (C) Weighted projection of the probe distribution as in (D) in a “flat” Bull's Eye representation.
(TIF)

Figure S6 **Probes' colocalization.** Probes' (Prosense-680, Clio-750) activity colocalization within the left ventricles for a ischemia reperfusion injury in wild type (IS), myocardial infarction in wild type (MI) and myocardial infarction in ApoE^−/−^ mouse. For each model the distribution of the molecular probes CLIO-750 and Protease-680 was measured, computed and then compared. We therefore calculated the cross correlation function (CCF) which provides information on how these probes correlate to each other spatially. (A) Full width at half maximum (FWHM) of the cross correlation function (CCF) in correspondence of its maximum along the x direction of the fitting ellipsoid's horizontal plane, correlating Prosense-680 and CLIO-750 3D distributions. (B) FWHM of the CCF in correspondence of its maximum along the y direction (orthogonal to x), correlating Prosense-680 and CLIO-750 3D distributions. (C) FWHM of the CCF in correspondence of its maximum and along the z direction (orthogonal to the x,y plane), correlating Prosense-680 and CLIO-750 3D distributions. (D) Average maximum values of the CCF correlating Prosense-680 and CLIO-750 3D distributions. The FWHM is the widest along all directions for the ischemic infarct mouse model, while it is the narrowest for the wt MI model indicating that for this model the probes are very well confined within a small volume. For the ApoE^−/−^ mouse model the FWHM appears to assume values in between the two other models suggesting that the inflammation (i.e. molecular probe activity) is more widespread than MI. High values of correlation (D) for all models suggest the two probes are significantly colocalized. Furthermore, the CCF maxima occur for relative distributions' translations equal to zero. Such information endorse the fact that the two signals are highly spatially correlated.
(TIF)

Text S1 **Supplemental Material.**
(DOC)
