# Supplementary figures and images for: Mapping Molecular Agents Distributions in Whole Mice Hearts Using Born-Normalized Optical Projection Tomography
Source: PLoS One. 2012 Apr 11;7(4):e34427. doi: 10.1371/journal.pone.0034427 (PMC3324534; doi:10.1371/journal.pone.0034427)

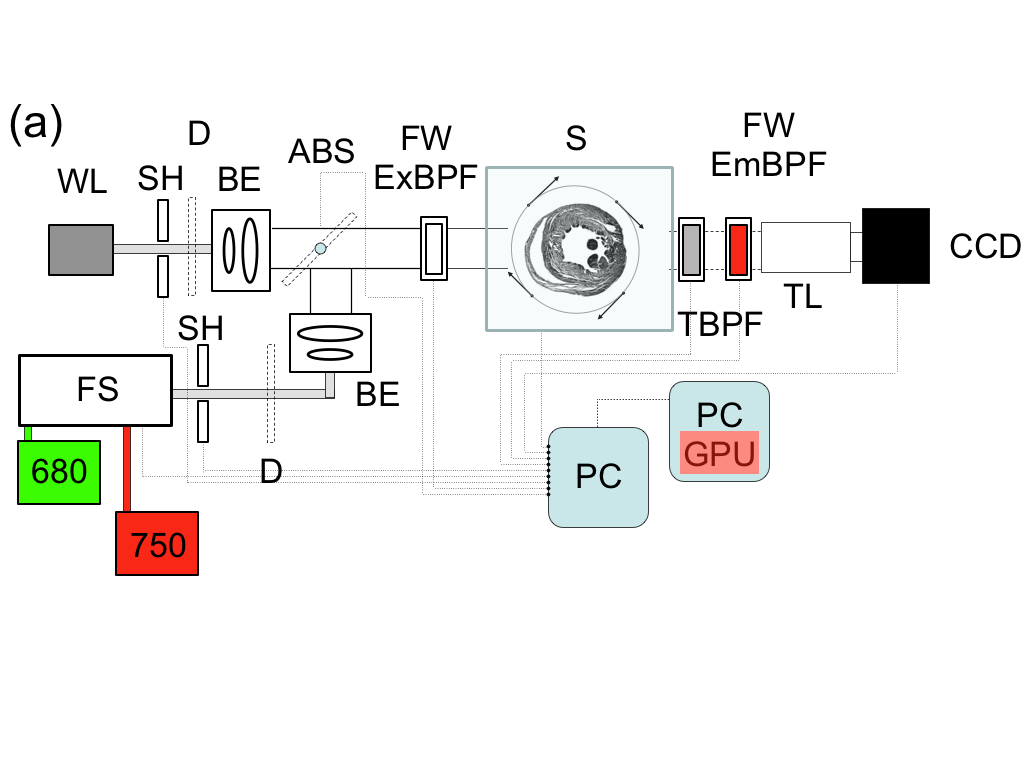

Supplement: Figure S1 — Experimental setup. (A) WL white light source, SH shutter, BE beam expander, D diffuser, FS optical fiber switch combined with a 680 nm and 750 nm wavelength excitation laser sources, FW ExBPF fast automated filter wheel with band pass filters for both excitation and transmission mode, FW ExBPF fast automated filter wheel with emission band pass filters and neutral density filters, ABS automated beam switcher, S sample, TBPF automated tunable bandpass filter, TL telecentric lens imaging system, CCD imaging camera. (TIF) [file pone.0034427.s001.tif]

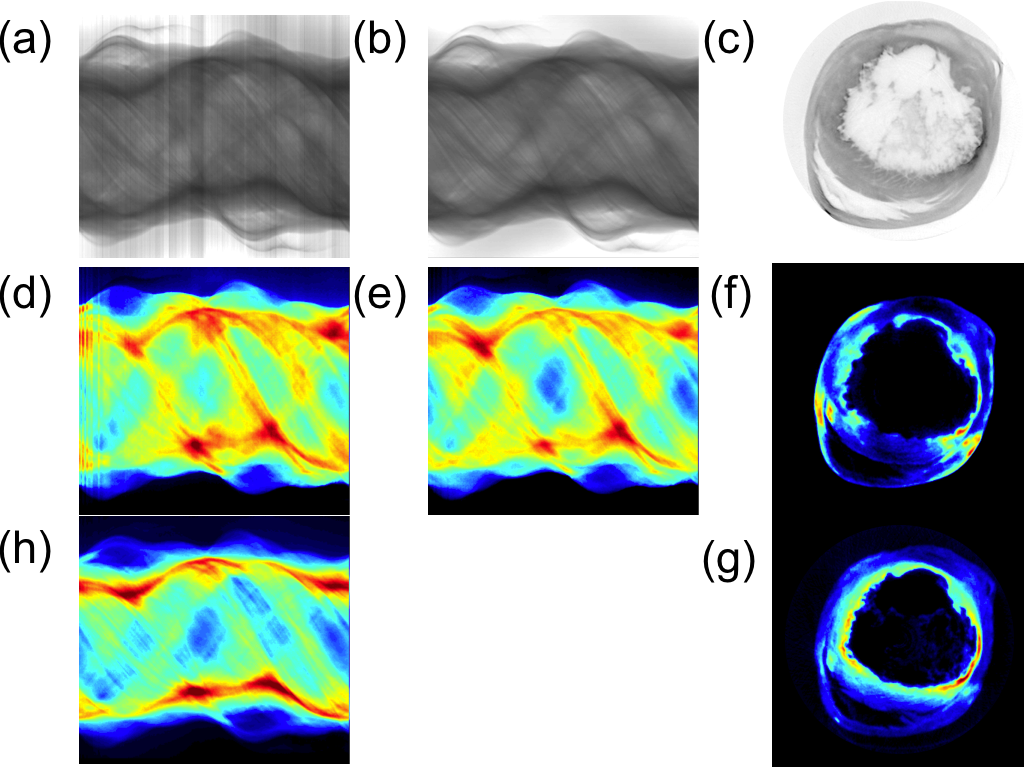

Supplement: Figure S2 — Power equalization and Born normalization. Absorption sinogram without (A) and with (B) intrinsic intensity fluctuations corrections. Lamp fluctuations are visible as vertical stripes in (A). (C) Reconstruction after power equalization. Fluorescence sinogram without (D) and with (E) excitation-source intensity fluctuations corrections with relative slice reconstruction (F). (H) Born-normalized fluorescence sinogram after intrinsic and excitation intensity correction with relative slice reconstruction (G). (TIF) [file pone.0034427.s002.tif]

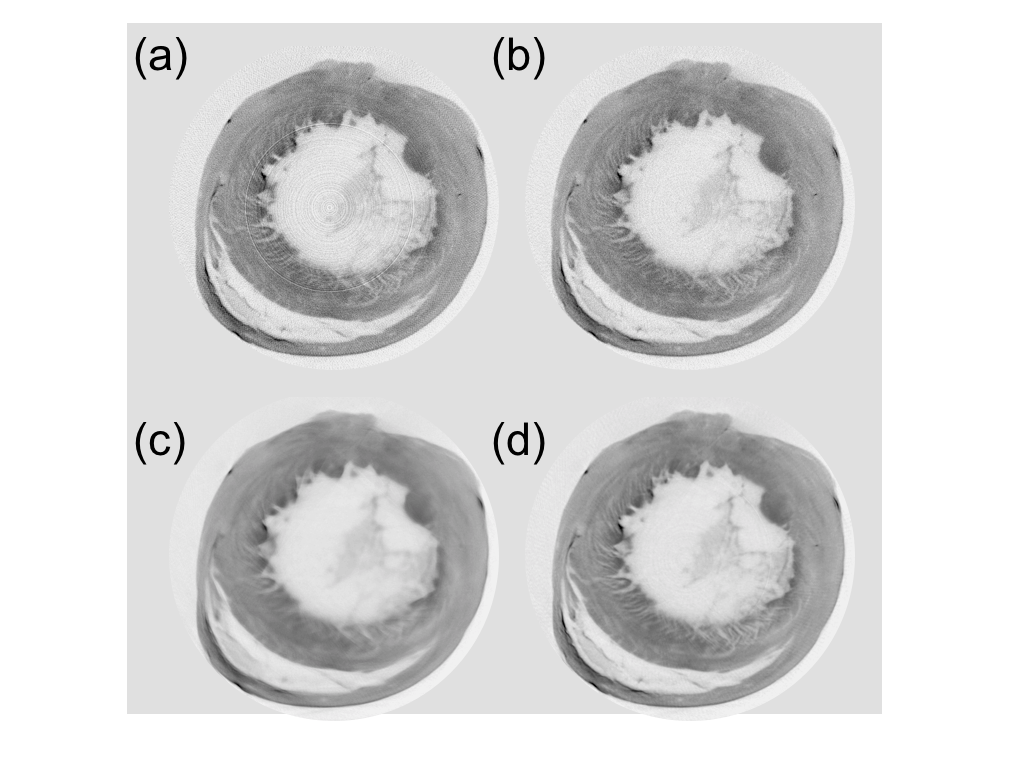

Supplement: Figure S3 — Ring artifacts and random noise reduction. (A) Axial reconstruction, raw data. (B) Reconstruction after post-processing for ring artifacts removal. (C) Reconstruction after ring and noise reduction (by 2D median filter). (D) Reconstruction after ring and noise reduction (by BM3D filter). Artifacts and random noise are corrected on projections, i.e. before backprojection transformation. (TIF) [file pone.0034427.s003.tif]

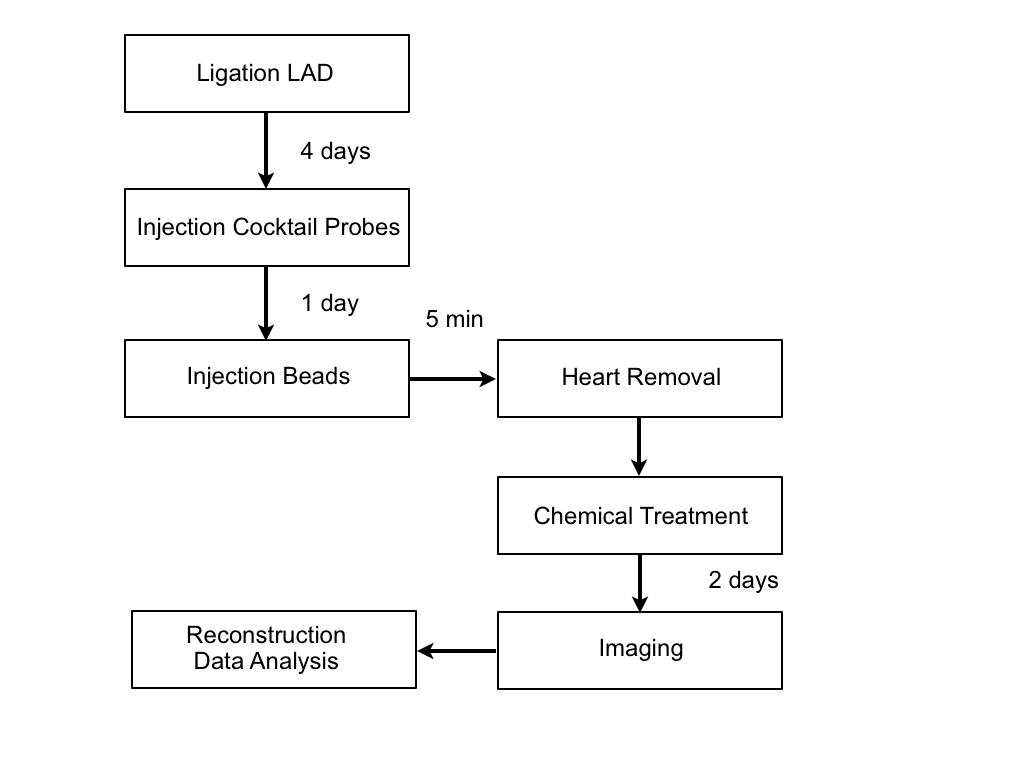

Supplement: Figure S4 — Experimental protocol. Four days after artery ligation, mice were intravenously co-injected with a cocktail of monocyte-targeting nanoparticle (CLIO-750), and a cathepsin B imaging probe (Prosense-680). After one day fluorescent microbeads were injected in order to delineate the infarct area. Heart was then removed after 5 minutes, chemically treated for 48 hours and imaged. During acquisition data were processed and reconstructed, and finally analyzed. (TIF) [file pone.0034427.s004.tif]

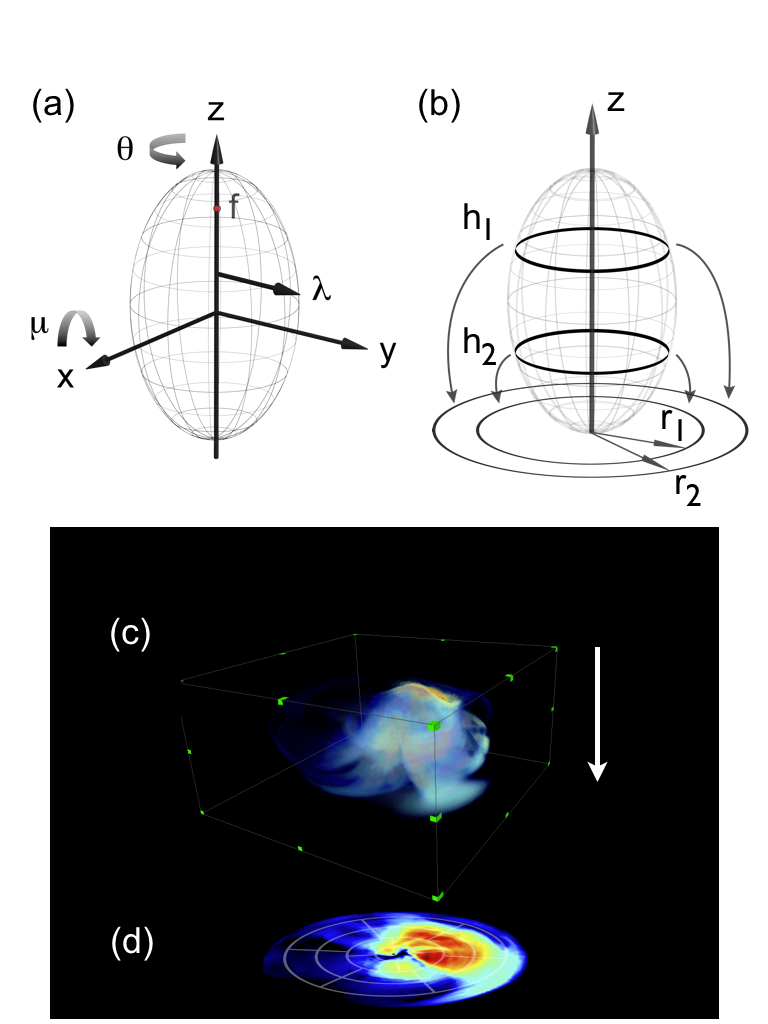

Supplement: Figure S5 — Bull's Eye representation. Cylindrical (A) and Bull's Eye (B) coordinate transformation. (C) Weighted projection of the probe distribution as in (D) in a “flat” Bull's Eye representation. (TIF) [file pone.0034427.s005.tif]

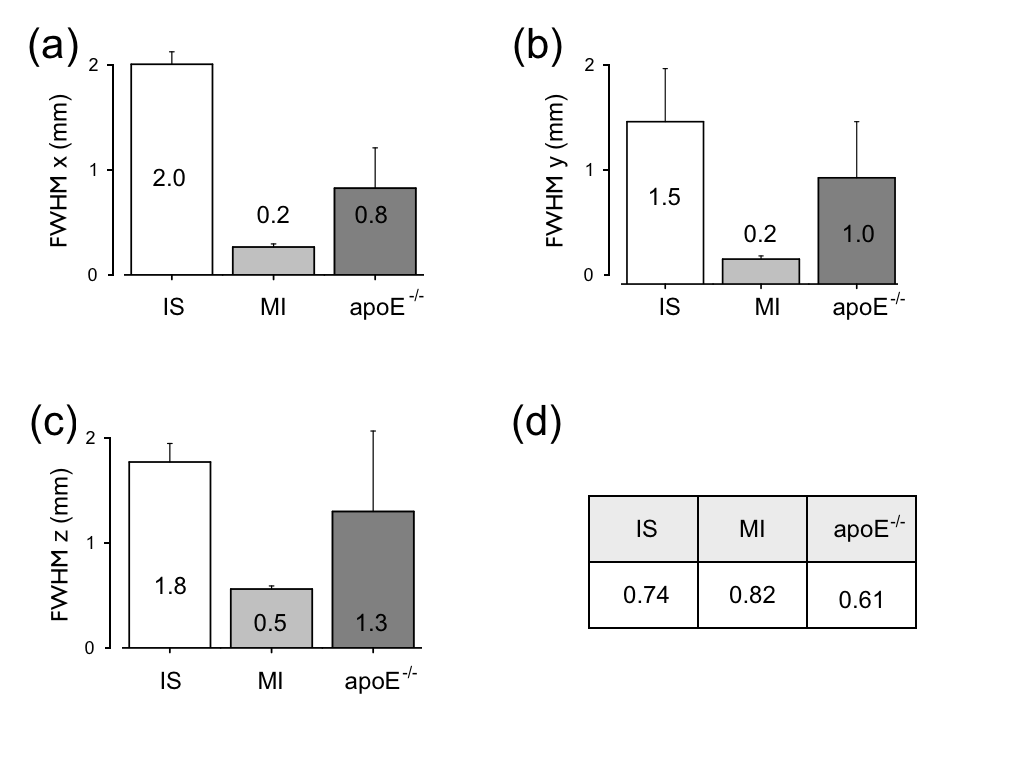

Supplement: Figure S6 — Probes' colocalization. Probes' (Prosense-680, Clio-750) activity colocalization within the left ventricles for a ischemia reperfusion injury in wild type (IS), myocardial infarction in wild type (MI) and myocardial infarction in ApoE−/− mouse. For each model the distribution of the molecular probes CLIO-750 and Protease-680 was measured, computed and then compared. We therefore calculated the cross correlation function (CCF) which provides information on how these probes correlate to each other spatially. (A) Full width at half maximum (FWHM) of the cross correlation function (CCF) in correspondence of its maximum along the x direction of the fitting ellipsoid's horizontal plane, correlating Prosense-680 and CLIO-750 3D distributions. (B) FWHM of the CCF in correspondence of its maximum along the y direction (orthogonal to x), correlating Prosense-680 and CLIO-750 3D distributions. (C) FWHM of the CCF in correspondence of its maximum and along the z direction (orthogonal to the x,y plane), correlating Prosense-680 and CLIO-750 3D distributions. (D) Average maximum values of the CCF correlating Prosense-680 and CLIO-750 3D distributions. The FWHM is the widest along all directions for the ischemic infarct mouse model, while it is the narrowest for the wt MI model indicating that for this model the probes are very well confined within a small volume. For the ApoE−/− mouse model the FWHM appears to assume values in between the two other models suggesting that the inflammation (i.e. molecular probe activity) is more widespread than MI. High values of correlation (D) for all models suggest the two probes are significantly colocalized. Furthermore, the CCF maxima occur for relative distributions' translations equal to zero. Such information endorse the fact that the two signals are highly spatially correlated. (TIF) [file pone.0034427.s006.tif]
