# Supplementary material for: Mapping Molecular Agents Distributions in Whole Mice Hearts Using Born-Normalized Optical Projection Tomography
Source: PLoS One. 2012 Apr 11;7(4):e34427. doi: 10.1371/journal.pone.0034427 (PMC3324534; doi:10.1371/journal.pone.0034427)
Supplement: Text S1 — Supplemental Material. (DOC) [file pone.0034427.s007.doc]

**SUPPLEMENTAL MATERIAL**

**Experimental setup.**

Near-infrared Born-normalized dual-probe molecular imaging optical projection tomography measurements were performed with the use of a modular home-built imaging system capable of acquiring both fluorescence and optical attenuation data in transillumination modes. The experimental setup is shown in detail in Figure S1. A white-light source beam (HL250-AY; AmScope) was collimated and a beam-expander with a combined two-lenses Galilean telescope (Thorlabs) and a diffuser (10DIFF-VIS; Newport) were employed in order to achieve uniform sample illumination over the camera’s field of view. White light was then spectrally filtered using two distinct narrow bandpass interference filters (Chroma) centered at 680nm and 750nm respectively with a 5 nm FWHM bandwidth each.

The filtered white light was used to acquire intrinsic transillumination images and ﬁlters with different optical densities (Thorlabs) were used in order to keep the CCD intensity values approximately 10% below saturation. Transillumination fluorescence images were obtained using two excitation laser diodes (BW-Tek) centered at 672nm and 748nm, tailored to the optical properties of VT-680 (Ex: 673nm , Em: 691nm) and VT-750 (Ex: 750nm, Em 780nm) respectively. The laser light was diffused and collimated in a similar way as the white light. Two narrow (5nm FWHM) bandpass interference filters centered at 680nm and 750nm respectively were used to remove any ASE component present in the excitation beam. A motorized fast change filter wheel (Thorlabs) and a shutter allowed for rapid filtering and path switching (intrinsic vs fluorescence).

The sample under investigation was hold in place in a cylindrical agarose holder immersed in a near-infrared (NIR) antireﬂective-coated imaging chamber filled with a solution of benzyl alcohol and benzyl benzoate (BABB) and rotated along its vertical axis. 360° rotation was provided by a stage controller (Newport, PR50) with an absolute accuracy of 0.05°. Manual controllers provided for tilt adjustment of the vertical axis while an automatic shutter prevented light exposure during rotation, reducing photobleaching of the molecular contrast agents.

The imaging system collected photons in transillumination mode by way of a telecentric optical setup. Photons were detected by an ultralow-noise, cooled charge-coupled device (CCD) camera (VersArray, Princeton Instruments) with a 1024x1024 or 512x512 (after binning) sensor element.

The exposure times were set to 0.1s for the translumination channels and 1s or 3s for the fluorescent channels (VT-680 and VT-750 respectively). All filter wheels and shutters could be fast interchanged to switch between the fluorescence and the intrinsic acquisition modalities. Dual probe imaging was achieved by spectrally separating the signal and inserting in front of the imaging optics two different 40-nm-wide band-pass interference filters tuned on the emission maxima of the molecular probes (Andover, 710nm and 790nm, for VT-680 and VT-750 respectively). Multispectral transillumination absorption imaging was obtained by tuning an automated tunable filter (10 nm bandwidth) over the visible and near infrared part of the spectrum. All filter wheels, shutters, tunable filters, fiber switches, CCD, rotational stage, are controlled by a PC. A separate PC hosting a GPU is connected through Ethernet cable to the first one and used for data filtering, noise reduction, backprojection.

**Transillumination OPT and backprojection algorithm.**

Under the approximation of negligible scattering and through the use of optical imaging systems with high telecentricity, transmission OPT can be considered as the optical analogue of X-CT. The mathematical problem can therefore be solved in a similar fashion using the parallel beam backprojection (FBP) algorithm while the concept of projection and tomographic reconstruction can be introduced through the use of the Radon transform. Let define our imaged object *f(x,y)* where *f* describes the image density distribution, i.e. the spatial distribution of the absorption coefficient *μ*_a_ within our sample at the illumination wavelength λ. From the Radon theory the line integral of the object along the line (projection direction) *L* which forms an angle *θ *with the *x*-axis is defined as the Radon transform

where s is the coordinate in the two-dimensional detection plane.

For a particular choice of projection angle *θ_0_*, the integral is defined as the “projection” of the object *f* along the *L* direction. The array of all possible projections is defined as the *sinogram* of the object *f.* In practice a finite number of projection angles is considered, with *θ* usually spanning a half or full rotation angle with 1 degree step. The coordinate *s* in the detection plane is usually indexed along 512 or 1024 detection points and represents the number of pixels along the horizontal direction of the CCD camera. In OPT one can describe each ray emitted from a distinguished source *x*_s_ in a discretized one-dimensional fashion as traveling through the object *f* and being collected on the CCD at the detector position *x*_d_. In this case the relationship between the source and the collected signal is easily expressed in one dimension by the exponential Beer-Lambert type integral:

$I=I_{0}e^{-\int\mu_{a}(l)dl}$,

where *I_0_* is intensity of the source *x*_s_, *I* is light intensity detected at pixel *x*_d_ on the CCD camera, and $l$ is position along the projection direction *L*. In transillumination OPT the object *f(x,y)* corresponds therefore to the two-dimensional absorption map *μ*_a_(*x,y*) and the reconstruction problem consists in computing the absorption map given all the projections . This can be done through the use of the filtered backprojection method (FBP). Finally, since the intensity measurements correspond to exponentially attenuated light, the measured data has to be logarithmically transformed in order to obtain the actual values for the optical absorption coefficient. For a 3D object this approach can be naturally implemented by considering it as the combination of N planes orthogonal to the axis of rotation.

**Born-normalized reconstruction of fluorescence distribution**

Even though reconstructions of optical absorption with OPT could provide valuable high resolution information on tissue morphology, of particular interest is also the possibility to obtain high resolution tomographic information related to the distribution of fluorescent molecular agents. This option is very attractive in the way that allows to complement, at a different resolution scale, information previously collected with in vivo macroscopic molecular imaging techniques, such as FMT (Fluorescent Molecular Tomography) or MSOT (Mesoscopic Optical Tomography). In a discretized one-dimensional fashion, each ray at the excitation wavelength *λ*_ex_, emitted from an arbitrary source position *x*_s_, is exponentially attenuated as it passes through the imaged object. After exciting fluorochrome located at position *x*, the radiation at the emission wavelength *λ*_fl_ is filtered and collected into the corresponding pixel at virtual position *x* on a CCD camera using focused objective. In this case, the reconstructed values will rather correspond to the concentration of fluorescent agent in tissue instead of distribution of the optical absorption coefficient. Unfortunately, most organs present a varying spatially dependent level of absorption making incorrect the direct use of the Radon algorithm for backprojecting the fluorescence images. Therefore, here we use a Born ratio based on a normalized transillumination approach [1]. Transillumination in both fluorescence and absorption mode are collected over 360 degrees and the normalized Born intensity *U*_B_, defined as ratio of the measured fluorescence to the corresponding intensity at the excitation wavelength, is introduced as

$U_{B}(x_{s},x_{d})=\frac{U_{fl}(x_{s},x_{d})}{U_{ex}(x_{s},x_{d})}$. (1)

Once the correct Green’s functions for both the excitation and emission photons are defined, the tomographic set of Born normalized measurements can be then expressed as *U*_B_ = W*A*, where W represents the forward model matrix, describing the propagation of both intrinsic and fluorescence light through the imaged sample, and A is the unknown volumetric distribution of the fluorophores. The latter quantity can be finally obtained by applying the Radon backprojection to the set of measurements *U*_B_. Unlike the regular absorption OPT, which reconstructs optical absorption values, here the inversion can be directly performed on the measured data without applying the logarithmic function.

**Time dependent intensity fluctuations.**

For both transmission and fluorescence OPT, each recorded projection is proportional to the intensity of the illuminating source. In order to avoid artifacts in the final reconstructions the light source has to satisfy two constraints in terms of both spatial and time intensity distribution. The first condition is achieved with the combination of a beam expander coupled with a Galilean telescope and a diffuser, which contributed in making the incident light uniformly spatially distributed over the entire field of view of the sample. The second condition is related to time fluctuations (both short and long term) of the intensity of the illuminating light source. As possible sources, a white light (appropriately filtered), an LED or a laser, can be adopted but due to its narrow spectral properties a laser is more indicated in particular for fluorescence OPT. Independently of the choice all sources suffer of intrinsic intensity instabilities, which in the worst case amount up to 10%, and will affect the collected intensity of the projection data. As a consequence they will contribute to affect the sinograms’ quality and potentially lead to reconstructions’ smear artifacts.

These intensity instabilities can be compensated in different ways. Either with the use of a beamsplitter combined with a photodiode [2] or by following a much simpler approach (as proposed here) based on the total recorded light invariance for all projections by way of a post processing algorithm applied on the acquired data (Figure S2).

For clarity we limit the description to a single slice since the algorithm can be easily extended to the whole dataset. First the equalization of transmission mode OPT is given and then we will introduce the case for fluorescence Born-normalized OPT.

The transmission OPT equalization algorithm is based on the fact that the integral is constant for each * θ_0_  *∈ *θ. *Being θ-invariant, its value can therefore be used in the power equalization formula.

Now discretizing the acquired data and modeling the lamp fluctuations as zero-mean Gaussian randomly distributed, an estimation of can be calculated by way of:

,

where P is the total number of projections, *θ*_p_  indicates the angle of the *p*-th projection and *s*_k_indexes the CCD’s array of pixels for the slice (i.e. CCD’s line) under consideration. Obviously differs for each slice.

While power equalization can be directly applied to the transmission data, this is not possible when fluorescent data are considered, and a more sophisticated approach is required. In fact the condition of constancy for all the views in total fluorescence intensity is met only after Born-normalization.

For the case of emission OPT i.e. fluorescent mode, the object *f(x,y)*  represents the fluorophore activity distribution *f*_fl_ of the molecular imaging contrast agent. For this case the term cannot be considered constant over different projections views due to the presence of absorption for both excitation and emission light. In order for the term to be considered constant, has to be previously Born-normalized. Thus the algorithm differs from the transillumination mode because it requires the absorption information for normalizing the data.

**Ring artifacts removal.**

Ring artifacts are detrimental features, which worsen the quality of tomographic images such as X-ray CT as well as OPT. They basically arise due to the detectors non-linearity or drift responses and show up within the sinogram as straight lines along the angle direction. Very often these lines are barely above the noise level and cannot be not distinguished within the sinogram data, but the backprojection algorithm amplifies these feature making the artifacts visible on the reconstructed images with a characteristical ring shape centered around the center of rotation. A simple flat-field correction is rarely sufficient and different removal algorithms have been developed acting either on the sinogram data or on the reconstructed images. The first approach is usually more efficient than the second, and it was our method of choice (Figure S3)**.** Four steps were implemented:

1. First we calculated the “averaged sinogram signal” obtained by averaging the sinogram along the angular dimension. If we consider the measured projection as the sum of a “true” noise-free image with a noisy component (due to the detectors anomalies), this processing leads to a smooth signal (the average of n ‘real’ different projections) superimposed on sharp spikes that are due to the pixels’ anomalies.
2. Because these signal spikes are not smeared out due to the fact that they do not vary in position during sample’s rotation we then applied a smoothing filter to the averaged sinograms in order to separate the sharp components from the signal to be reconstructed.
3. We then computed the difference between the pre-filtered and the filtered signals and obtained the amount of correction to be applied on the raw data.
4. Finally we corrected each projection by subtracting the signal computed in step 3, and obtained the final reconstructions by backprojecting the treated data.

The detectors’ non-ideal behavior has been modeled as the sum of two distinct components: a random Gaussian-distributed component superimposed to all pixels, and a “salt and pepper” component superimposed only on certain pixels. The smoothing filter consisted of a two-cascade filters which first reduced the “salt and pepper” component by a 7 samples length median filter, and subsequently reduced the Gaussian component by applying an adaptive 7 samples length Wiener filter.

**Image noise removal**

Reduction of the random noise present in the reconstructions was achieved by way of a specific filtering scheme based on the BM3D algorithm [3] which has been demonstrated to be suitable for efficiently increasing the quality of severe noisy absorption OPT reconstructions [4].

Because in transillumination mode, OPT works at high light source intensities (i.e. high photon numbers) the noise is modeled as independent Gaussian, additive with zero mean and with unknown variance. Nevertheless this latter parameter can be accurately estimated by several methods directly on the noisy observations.

A noisy image *I_noisy_(x,y)* can be assumed to be the sum of the noise-free image *I(x,y)* plus the random camera noise *n(x,y)* respectively

(1)

where *x,y* are the image pixels coordinates. The BM3D filter works on the assumption that “real life” images are characterized by a high level auto-similarity. This property can be mathematically expressed by a function that gives the “distance” between image fragments. Fragments similar to others can be gathered into a 3D array and once transformed into the spectrum space can be well described as a combination of a few spectrum elements [5]. In this way the noise-free signal energy can be concentrated in a fewer number of coefficients leading to highly sparse signal representation.

BM3D achieves the sparsity by grouping and performs efficient noise reduction assuring important image features (such as details) preservation while introducing a very few image artifacts even after image transformations such as filtered backprojection FBP [4].

The grouping is performed by extracting an image fragment and comparing with other image fragments by using the Block Matching techniques. Similar fragments are collected into a 3D stack which is called group. Each group is transformed into a new space (e.g. Discrete Cosine Transform domain) where the noise free component is well represented by a few high magnitude coefficients. Being the noise statistical independent within a group (if no fragment overlapping occurs) and being white, its representation cannot be sparse, leading to low magnitude coefficients for the noise in that domain. The advantage of working in this manner is the easy signal-noise separation by thresholding. In fact, thresholding preserves the few high-magnitude transform coefficients (likely due to the noise-free signal) while discarding the low ones (likely due to the noise). This approach, called collaborative filtering, can be summarized into 5 main steps: grouping, 3D transformation of a group, shrinkage in the transformed domain, inverse 3D transform, and blocks replacement with a subsequent overlapping pixels weighted averaging. This process is usually repeated twice substituting the noise shrinkage step with a Wiener noise removal approach improving the filtering performance. Complete details of the algorithm can be found in Davov et al [3]. The BM3D filter in respect to others filtering schemes such as the median gives better results in terms of both visual and SNR evaluations when applied to absorption OPT projections before reconstruction (Figure S3)**.**

**Hardware acceleration for real time reconstructions.**

Speed-up of the processing time required for image reconstructions was facilitated via hardware acceleration [6]. A Radon backprojection algorithm was implemented on an NVIDIA Tesla C1060 graphic card by taking advantage of the massive amount of similar calculations that needed to be computed and by distributing them on different multiprocessors. This technology is equipped with 240 streaming processors at 1.3 GHz, 4GB GDDR3 onboard memory and 102 GB/s bandwidth memory access per GPU unit. The card is a low cost - high-performance computing (HPC) system capable of 933 GFLOPs/s of processing performance. CUDA technology permits to program the card using the unified design of the NVIDIA graphics-processing unit [7,8] and the GPUs are handled as a coprocessor executing data-parallel kernel functions [9]. “NVCC” NVIDIA compiler and “CUFFT” (CUDA for Fast Fourier Transform) together with C and C++ programming languages were the tools used for the software implementation.

We have implemented two different strategies for tomographic reconstructions: post processing and on the fly approach. The first method requires the acquisition of all projections stacked together. Each axial section of such a stack represents the sinogram of the final 3D volume’s slice. Because the backprojection algorithm is acting only on data from one slice at a time, parallel processing can be used to reconstruct different slices simultaneously. A full rotation of the sample is performed leading to a redundancy of the data referred to 180^0^ shifted projections. This symmetry allows reducing noise, compensating optical distortions and optimizing the algorithm in terms of speed. 180^0^ shifted data are flipped around the center of rotation and summed to the correspondent projection. All rows of each sinogram are treated with a Ram-Lak filter multiplied by a Hamming window in the Fourier transform domain. The backprojection algorithm is then applied to the filtered sinogram and the resulting reconstruction is placed into the final 3D array. Depending on the number of the axial reconstructions, code instructions are mapped to the CUDA kernel repeating the computations for all rows. Further speed optimization can be achieved with data pre-allocation into the Tesla memory, e.g. instance frequency filter coefficients sinuses and cosines of rotation angles, spatial coordinates for data backprojection and other required arrays. Such processing step is computed only once, while sinograms’ mapping to complex format, FFT, frequency filtering, IFFT, backprojection - are treated in parallel (as kernels) on-board by the Tesla card**.** The reconstructed stack of slices is then moved back to host.

The second approach is implemented for on-the-fly reconstructions. During acquisition, each single projection is immediately processed and the entire volume is kept in the Tesla memory during the entire measurement experiment. This approach is more memory consuming and it can be used only for smaller projection size (i.e. 512x512) while for bigger projections such as 1024x1024 it requires more than the 4GByte available on the card, if processed in floating point precision. A further optimization is represented by the filtering operation in the spatial domain instead of the frequency by convolution integral. A short-length filter (21 coefficients) was tested among the dataset but its use was restricted to only 1024^3^ reconstruction volumes.

**Bull’s Eye representations.**

Another very useful segmentation representation of the LV due to its broad use in cardiovascular imaging and recommended by the American Heart Association (AHA) is the Bull’s Eye representation. According to AHA nomenclature the whole myocardium can be divided in 17 distinct sectors [10] (Figure 6B). Planar Bull’s Eye maps of the LV are generated by orienting the main axis of the LV along the vertical direction and projecting on the horizontal plane passing through the heart’s apex, all the circular sections of the ellipsoid. The circles are then mapped on the horizontal plane with a radial distance *r*_i_ from the apex given by their planar distance *h*_i_(vertical) from the apex **(**Figure S5**).** The diameter of the Bull’s Eye plot will therefore be equal to the length of the main axis of the ellipsoid. As mentioned for the cylindrical maps, because the myocardial walls have a significant thickness with respect to their ellipsoid’s axes, multiple Bull’s Eye plot exist for each concentric ellipsoid. Even though 3D representations are straightforward to obtain, we opted again for a 2D visualization (“2D Bull’s Eye total projection”), integrating the total signal along the radial direction of the ellipsoid.

1. Vinegoni C, Razansky D, Figueiredo JL, Nahrendorf M, Ntziachristos V, Weissleder R. Normalized Born ration for fluorescence optical projection tomography. Opt. Lett. 2009;34:319-21.

2. Walls J, Sled J, Sharpe J, and Henkelman R. Correction of artefacts in optical projection tomography. *Phys. Med. Biol.* 2005;*50*:4645–4665.

3. Dabov K, Foi A, Katkovnik V, and Egiazarian K. Image denoising by sparse 3D transform-domain collaborative filtering. *IEEE Trans. Image Process.* 2007;16:2080-2095.

4. Feruglio FP, Vinegoni C, Gros J, Sbarbati A, Weissleder R. Block matching 3D random noise filtering for absorption optical projection tomography. *Phys. Med. Biol.* 2010;55:5401-15.

5. Katkovnik V, Foi A, Egiazarian K, Astola J. From Local Kernel to Nonlocal Multiple-Model Image Denoising. Int. J. of Comput. Vis. 2010;86:1-32.

6. Vinegoni C, Fexon L, Feruglio FP, Pivarov M, Figueiredo JL, Nahrendorf M, Pozzo A, Sbarbati A, Weissleder R, Opt. Exp. 2009;17:22320-32.

7. Nickolls J, Buck I, Garland M, and Skadron K, Scalable parallel programming with CUDA. Queueing Syst. 2008;6;40–53.

8. Nickolls J, and Buck I. NVIDIA CUDA software and GPU parallel computing architecture. Microprocessor Forum 2007.

9. Ryoo S, Rodrigues CI, Stone SS, Baghsorkhi SS, Ueng SZ, Stratton JA, and Hwu WW. Program Optimization Space Pruning for a Multithreaded GPU. Proceedings of the sixth annual IEEE/ACM international symposium on Code generation and optimization, April 05-09, 2008, Boston, MA, USA

10. Cerqueira M, Weissman N, Dilsizian V, Jacobs A, Kaul S, Laskey W, Pennell D. Standardized myocardial segmentation and nomenclature for tomographic imaging of the heart: a statement for healthcare professionals from the Cardiac Imaging Committee of the Council on Clinical Cardiology of the American Heart Association. *Circulation*, 2002;*105*:539-542.
